# Formulation of water-soluble *Buddleja globosa* Hope extracts and characterization of their antimicrobial properties against *Pseudomonas aeruginosa*

**DOI:** 10.3389/fphar.2022.921511

**Published:** 2022-11-10

**Authors:** Nicolas Araya, Martín A. Leiva-Soto, Maria V. Bruna, Almendra Castro-Munoz, Beatriz Behrend-Keim, Daniel Moraga-Espinoza, Tania F. Bahamondez-Canas

**Affiliations:** ^1^ Escuela de Química y Farmacia, Facultad de Farmacia, Universidad de Valparaíso, Valparaíso, Chile; ^2^ Centro de Investigacion Farmacopea Chilena, Universidad de Valparaíso, Valparaíso, Chile

**Keywords:** *Buddleja globosa*, natural extracts, phytopharmaceuticals, antimicrobials, *Pseudomonas aeruginosa*

## Abstract

*Buddleja globosa* Hope (BG) extracts are traditionally used to treat skin and gastric ulcers due to their healing properties. Non-aqueous solvents such as ethanol and DMSO are usually used to extract naturally occurring compounds. However, the cytotoxicity of these solvents and the low water solubility of the extracted compounds can hinder their biomedical applications. To overcome the limited solubility of the BG extracts, we aimed to enhance the solubility by processing a standardized hydroalcoholic extract (BG-126) through spray drying (SD), with and without two solubility enhancers. Spray-dried BG (BG-SD) extracts and spray-dried BG extracts plus polyvinylpyrrolidone (BG-SD PVP) and Soluplus^®^ (BG-SD SP) were developed starting from BG-126 (containing 53% ethanol). These four formulations were characterized by total phenolic content, water solubility at 25°C and 37°C, and antimicrobial properties against *Pseudomonas aeruginosa*. All the SD formulations presented a solubility that allowed them to reach maximum concentrations of 1,024 μg/ml catechin for BG-SD and 2,048 μg/ml catechin for BG-SD PVP and BG-SD SP for antimicrobial testing. BG-SD showed the highest antimicrobial potency with a minimum inhibitory concentration (MIC) of 512 μg/ml catechin, followed by BG-126 with a MIC of 1,024 μg/ml catechin and SP. BG-126 was also shown to inhibit biofilm formation, as well as the excipients PVP and SP. The spray-dried BG (BG-SD) extract represents a promising natural active component with enhanced antimicrobial properties against *P. aeruginosa* for further research and the development of novel phytopharmaceuticals.

## 1 Introduction

*Pseudomonas aeruginosa* is an opportunistic pathogen commonly isolated from complex wounds (Kirketerp-Moller et al., 2008). Despite the efforts in wound care, chronic wounds remain a clinical challenge due to their susceptibility to complications such as infection or reoccurrence that negatively impact the life quality of these patients. While there is no clear cause of the chronicity of wounds, several identified factors that contribute to this state range from metabolic, nutritional, and local factors (Han and Ceilley, 2017).

Evidence shows that microbial biofilms colonize more than 60% of chronic wounds, and *P. aeruginosa* is one of the most predominant microorganisms (James et al., 2008). Also, colonization by this pathogen is related to larger ulcer sizes and more healing complications (Danielsen et al., 1998; Kirketerp-Moller et al., 2008; Malone et al., 2017). Biofilms are microbial communities that grow adhered to surfaces, protecting themselves from the environment with a self-secreted extracellular polymeric substance (EPS). This EPS, also known as the biofilm matrix, shields the community from external factors such as shear stress, antimicrobial agents, and immune factors (Flemming and Wingender, 2010). Therefore, when these biofilms develop on the surface of wounds, they contribute to a chronic state of inflammation that delays wound healing (Bjarnsholt, 2013). Although biofilms are commonly found in nature, biofilm colonization usually occurs in the tissues of patients affected by underlying diseases such as diabetes or venous ulcers (Wolcott and Ehrlich, 2008; Bjarnsholt, 2013).

As antimicrobial resistance to antibiotics increases, the World Health Organization has made several calls to promote the research and development of new antimicrobial agents, particularly focusing on a detailed list of pathogens (Taconelli and Magrini, 2017). *P. aeruginosa* is included in this list as a pathogen of high priority. In this scenario, natural extracts can still be envisaged as a source of novel antimicrobial compounds.

*Buddleja globosa* Hope (BG) is a native shrub from Chile, Perú, and Argentina, traditionally used to treat skin and gastric ulcers for its analgesic, anti-inflammatory, and antioxidant properties (Backhouse et al., 2008; Bustamante et al., 2015). Also, the antimicrobial properties of several components of BG have been reported against *P. aeruginosa*, *S. aureus*, and *E. coli,* among other microorganisms. Some examples of these compounds are catechin (Zhang et al., 2021), apigenin and its derivatives (Kim et al., 2020), carvacrol (Bhardwaj et al., 2019), verbascoside (Avila et al., 1999), and quercetin (Jaisinghani, 2017a). However, the antimicrobial activity of *B. globosa* extracts against *P. aeruginosa* proliferation and biofilm formation has not been evaluated.

On the other hand, natural extracts are usually obtained using non-aqueous solvents like ethanol, methanol, or DMSO. However, the low solubility of the extract components can hinder their biomedical application in the treatment of chronic or infected wounds as these solvents may exert cytotoxic effects against fibroblasts and keratinocytes (Singh, 2017; Calderon-Montano et al., 2018; Kar et al., 2021). Therefore, increasing the water solubility of natural extracts may maximize their efficacy and minimize or discard the cytotoxicity induced by non-aqueous solvents.

Different strategies have been studied to address these problems, like using nanocarriers and other solubilizing agents (Vanti, 2021). Soluplus^®^ is a graft copolymer of polyvinyl caprolactam, polyvinyl acetate, and polyethylene glycol, capable of solubilizing hydrophobic compounds by forming polymeric nanomicelles (Pignatello et al., 2022). Polyvinylpyrrolidone (PVP) is a versatile excipient useful for encapsulating hydrophilic and hydrophobic compounds in solid dispersions (Kurakula and Rao, 2020). The amphoteric nature of Soluplus^®^ has proven to enhance the solubility of poorly soluble natural extracts. Rajakumari et al. (2020) used Soluplus^®^ for grape seed extract processing by freeze drying and then tested the antioxidant activity of the formulation. This excipient has also been tested for isolated natural compounds like thymoquinone (TQ), a potent antineoplastic component extracted from *Nigella sativa* essential oil. TQ has low aqueous solubility which limits its further applications. Bergonzi et al. (2020) formulated TQ using an association of polymeric solubilizers, where Soluplus^®^ increased 10 times its solubility. This improvement is also related to an increase in its bioactivity against cancer cell migration.

Therefore, this research aimed to explore the antimicrobial properties of BG extracts against *P. aeruginosa* and to develop BG formulations from a hydroalcoholic extract with improved water solubility to enhance its antimicrobial potency without the need for ethanol as a solvent. To enhance the solubility of the BG extract, we developed three dry powder formulations of BG through spray drying (SD), starting from a standardized hydroalcoholic extract (BG-126) with and without two pharmaceutical excipients (PVP and Soluplus^®^). Then, we evaluated their antimicrobial properties against *P. aeruginosa*, an opportunistic pathogen commonly isolated from chronic wounds.

## 2 Materials and methods

### 2.1 Materials

The standardized ethanolic extract of *Buddleja globosa* Hope (BG-126) was kindly donated by Laboratorios Ximena Polanco (Santiago, Chile). Soluplus^®^ and Kollidon^®^ L30 (PVP) were gifted by BASF Chile. Crystal violet, methanol, acetic acid, MOPS buffer, fluorescein diacetate (FDA), tobramycin sulfate, glycerol, catechin hydrate, LB broth and agar, and phosphate-buffered saline (PBS) were provided by Sigma-Aldrich (St. Louis, MO, United States). Ethanol absolute was obtained from Millipore (Merck KGaA, Darmstadt, Germany), and acetone was obtained from J. T. Baker (Radnor, PA, United States). Verbascoside was provided by MedChemExpress LLC (Monmouth Junction, NJ, United States).

### 2.2 Spray drying

The hydroalcoholic BG-126 extract [53% (v/v) ethanol] was processed by SD, as received from the provider to obtain BG-SD. Two solutions were prepared with BG-126 and excipients. In total, three formulations were developed, as shown in Table 1. These solutions were spray-dried in a Büchi B-290 Mini Spray Dryer (Büchi Labortechnik AG, Flawil, Switzerland). After the drying process, the obtained formulations were stored in amber bottles inside a desiccator until use.

**TABLE 1:** BG formulations studied and prepared by spray drying.

Formulation	Description	Composition	SD parameters	Mass recovered (g)
BG-SD	Spray-dried *B. globosa* ethanolic extract	50 ml extract	IT: 90°C/OT 60°C	1.40
			A 29,75 m^3^/h	
			AF 474 L/h	
			F 6 ml/min	
BG-SD SP	Spray-dried *B. globosa* ethanolic extract + Soluplus^®^	50 ml extract +9.19 g of Soluplus^®^	IT 90°C and 120°C/OT 60°C	7.03
			A 29,75 m^3^/h	
			AF 474 L/h	
			F 6 and 3 ml/min	
BG-SD PVP	Spray-dried *B. globosa* ethanolic extract + PVP	50 ml extract +9.14 g PVP	IT 90°C/OT 60°C	9.42
			A 29,75 m^3^/h	
			AF 474 L/h	
			F 6 ml/min	

SD, spray drying; IT, inlet temperature; OT, outer temperature; A, aspirator; AF, atomization gas flow; F, flow of the feeding solution.

### 2.3 Characterization of the *Buddleja globosa* Hope formulations

#### 2.3.1 Chemical analysis

All the formulations (BG-126, BG-SD, BG-SD SP, and BG-SD PVP) were characterized according to their total phenolic content expressed as catechins using the Folin–Ciocalteu (FC) method, commonly used to analyze natural products (Chen et al., 2015). For determining the calibration curve, 10.9 mg of catechin hydrate was added to 50-ml volumetric flasks, and the volume was completed with distilled water. Then, five standard solutions were prepared from this stock solution at 10, 20, 30, 40, and 50 ppm in 10-ml volumetric flasks. Then, 0.5 ml of each standard solution was combined and vortexed with 2.5 ml of 10% FC (v/v). After 8 min of vortex mixing, 2.0 ml of 7.5% Na_2_CO_3_ (w/v) was added, and the mixture was allowed to sit for 1 h protected from light before recording the absorbance at 765 nm. After plotting the absorbance *versus* catechin concentration, a value of *R* ^2^ = 0.9927 was obtained. Finally, the following samples of the extracts were analyzed: 4.2 mg of BG-SD, 20.6 mg of BG-SD PVP, and 20.1 mg of BG-SD SP were prepared in 25-ml volumetric flasks, while 266 µl of BG-126 was diluted in a 100-ml volumetric flask. These solutions were analyzed by the FC method, and the catechin content was calculated by linear regression.

BG-126 and BG-SD were additionally characterized due to their verbascoside content using a Shimadzu HPLC system [SIL-20AC HT autosampler, LC-20AD pump, CTO-20AC column oven, DGU-2045 degasser, and SPD-M20A photodiode array detector (PDA)]. Separation was achieved on an Inertsil^®^ ODS-3 column (C18, 4.6 × 250 mm, 5 μm; GL Sciences Inc., Tokyo, Japan) at a flow rate of 1.0 ml/min using a binary gradient with water containing 5% phosphoric acid (mobile phase A) and acetonitrile (mobile phase B). The column oven was set to 30°C, and the PDA detector was operated at 330 nm. Peaks were recorded and integrated using LabSolutions DB (Shimadzu Corporation, Kyoto, Japan). A calibration curve of a verbascoside standard (99.83% purity) was prepared as follows: 8.4 mg of verbascoside was diluted in a 50-ml volumetric flask using 50% ethanol (v/v). A total of five 10 ml standard solutions were prepared from 8.4 to 167.7 mg/ml. These solutions were injected (20 µl) into the instrument. The calibration curve obtained had a value of *R* ^2^ = 0.9999. Then, 0.1 ml of BG-126 was diluted to 5 ml, and 4.5 mg of BG-SD was diluted to 25 ml in volumetric flasks. BG-SD PVP and BG-SD SP were not analyzed by this method due to their high polymeric content.

#### 2.3.2 Solubility and flowability

To determine the solubility of the SD formulations, 200 mg of BG-SD, BG-SD PVP, and BG-SD SP were weighed in 200-ml beakers. Then, known volumes of distilled water were added at 15 min intervals while mixing on a magnetic stirrer at 25°C until complete dissolution was observed, followed by the FC assay described previously. This test was also performed at 37°C considering further conditions used in bacterial culture.

The angle of repose was assessed using a glass funnel loaded with 1 g of the SD formulations at 6 cm from the surface. The angles of repose were captured using a monochromatic camera (Flea3^®^, FLIR Integrated Imaging Solutions, Inc.) and calculated by ImageJ software. The flowability of the dry powder formulations was described from “excellent” to “very, very poor,” as described by chapter 1,174 of the USP.

### 2.4 Antimicrobial properties against *Pseudomonas aeruginosa*

#### 2.4.1 Bacterial culture

*Pseudomonas aeruginosa* ATCC 27853 (Microbiologics, Inc., St. Cloud, MN, United States) was maintained in 50% glycerol stocks at −80°C. Fresh agar cultures were prepared in LB agar for each experiment starting from frozen stocks, and then, the plates were incubated overnight in a static incubator at 37°C (Heratherm IGS60, Thermo Fisher Scientific, Columbus, OH, United States). Then, bacterial suspensions were prepared in LB medium and adjusted to 3 × 10^7^ CFU/ml by spectrophotometry (at 600 nm, Synergy H1M, BioTek Instruments, Santa Clara, CA, United States). For the broth microdilution method, this suspension was diluted in 1:100 dilutions and added to tissue culture-treated 96-well plates (Corning Inc., Corning, NY, United States). For biofilm formation, 100 µl of the adjusted suspension (3 × 10^7^ CFU/ml) was added to black, flat-bottomed, tissue culture-treated 96-well plates (Corning Inc., Corning, NY, United States). After 1.5 h of incubation at 37°C and 75 rpm, we discarded the supernatant and rinsed the wells twice with 100 µl of sterile PBS. Finally, 200 µl of LB broth was added before a second incubation at 37°C and 75 rpm for 24 h.

#### 2.4.2 Determination of the minimum inhibitory concentration of the formulations in *Pseudomonas aeruginosa*

The formulations were tested using the broth microdilution method (EUCAST, 2003). Briefly, the formulations were prepared in 96-well plates and diluted in LB broth in serial two-fold dilutions, reaching a final volume of 100 µl of each concentration at 2x. Then, 100 µl of the bacterial suspension previously described was added to the multiwell plate with the treatment solutions to obtain the final concentrations ranging from 0.5 to 1,024 μg/ml catechin. The plates were incubated at 37°C and 180 rpm for 24 h before recording the absorbance at 625 nm as a measurement of bacterial proliferation. Tobramycin sulfate (1 μg/ml) was used as a positive control. Sterile formulation blanks were also included. Results were reported as %proliferation with respect to the untreated bacterial suspensions (negative control).

#### 2.4.3 Determination of the effect of the formulations on *Pseudomonas aeruginosa* biofilm formation

The three highest concentrations of the formulations (around the determined MICs) were tested against *P. aeruginosa,* as described previously. After 24 h of incubation, the supernatants were discarded and the plates were thoroughly rinsed with distilled water and air-dried over a paper towel. Then, 200 µl of methanol was added for biofilm fixation, followed by an incubation of 30 min at room temperature. Then, methanol was discarded, and the plate was allowed to air-dry overnight. A measure of 200 µl of 0.1% (w/v) crystal violet solution was added to each well of the plate for 15 min at room temperature. After rinsing with distilled water and air-drying, the retained crystal violet was dissolved with 30% (v/v) acetic acid for 30 min before a step of linear shaking for 10 s and recording the absorbance at 570 nm. The results were reported as %biofilm biomass using untreated *P. aeruginosa* as a negative control.

### 2.5 Statistical analysis

For bacterial proliferation and biofilm biomass determination, each condition was tested in quadruplicate in at least two independent experiments. Results were expressed as the mean ± standard deviation, and significant differences (*α* = 0.05) were analyzed by Tukey–Kramer honestly significant difference (HSD) test by JMP 10.0.0 software (SAS Institute Inc.).

## 3 Results

### 3.1 Total phenolic content and solubility of the *Buddleja globosa* Hope formulations

BG-126 and the three SD formulations were analyzed for total phenolic content using catechin as the standard. For BG-126, we determined a total phenolic content of 7,378 ± 45 μg/ml catechin that was close to that reported in the certificate of analysis of BG-126 (7,551 μg/ml catechin). On the other hand, the BG formulations developed by SD showed total phenolic contents from about 3.4% to 16.9% (Table 2). The lowest catechin content in BG-SD PVP and BG-SD SP compared to BG-SD was expected since the excipients added more volume to the powder formulations. We also determined the yield of the spray drying process using the catechin content of BG-126 as a reference. All formulations were prepared by spray drying 50 ml of BG-126 with or without the addition of excipients that contain 369 mg of catechin. The yield of the process was high, ranging from 64 to 87%.

**TABLE 2:** Characterization of BG formulations.

Formulation	Type of formulation	Catechin		Verbascoside		SD yield(expressedas catechin)	Solubility (expressed as µg/ml catechin)	
		% (w/w)	(µg/ml)	% (w/w)	(µg/ml)	%	25°C	37°C
BG-126	Solution	—	7,378	—	3,696	—	—	—
BG-SD	Dry powder	16.9	—	11.0	—	64.0	132.7	1,024
BG-SD SP	Dry powder	3.4	—	—	—	66.4	176.4	2,048
BG-SD PVP	Dry powder	3.5	—	—	—	87.0	175.0	4,116

BG, *Buddleja globosa* Hope extract; SD, spray-dried; PVP, polyvinylpyrrolidone; SP, Soluplus^®^

All the SD formulations showed poor flowability. The powder flow fluctuates from passable (BG-SD SP and BG-SD PVP) to poor (BG-SD) with angles of repose of 42, 44, and 50, respectively. The SD formulations as a total mass were very slightly or slightly soluble (˂10 mg/ml) at 25°C and soluble at 37°C. When comparing these solubilities expressed as µg/ml catechin, BG-126 retains the highest solubility as a hydroalcoholic extract, followed by the aqueous extracts BG-SD PVP, BG-SD SP, and BG-SD with the lowest solubility (Table 2). The solubility at room temperature improved slightly with the addition of excipients from 132 to 175 μg/ml catechin. PVP improved the solubility at 37°C, reaching a maximum of 4,116 μg/ml catechin (about 56% of the content of BG-126).

### 3.2 Determination of the minimum inhibitory concentration of the *Buddleja globosa* Hope formulations in *Pseudomonas aeruginosa*

**FIGURE 1:**
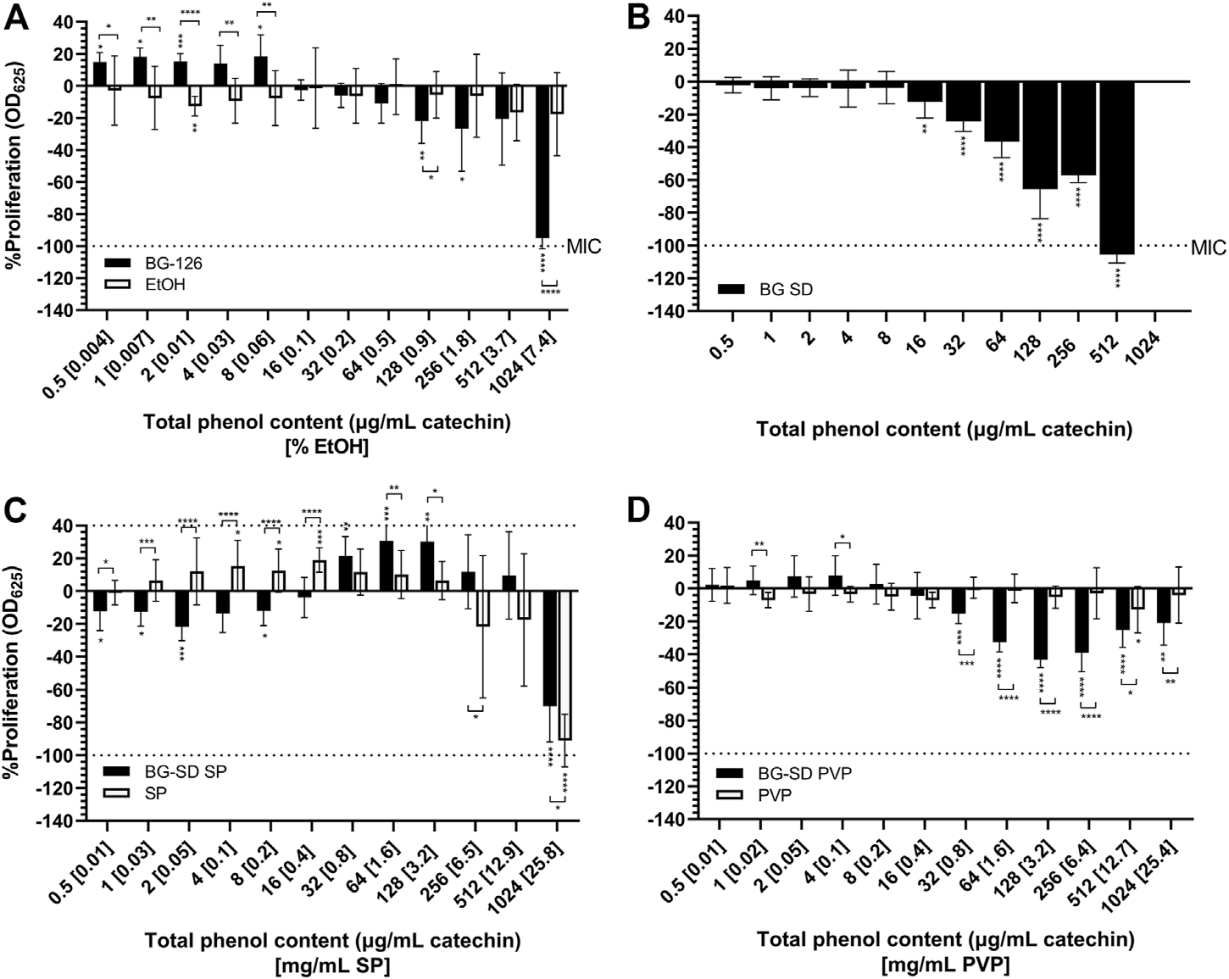
Effect of *Buddleja globosa* Hope (BG) extracts on the proliferation of *Pseudomonas aeruginosa*. *P. aeruginosa* was treated for 24 h with a hydroalcoholic BG extract (BG-126) **(A)**, spray-dried BG extract (BG-SD) **(B)**, spray-dried BG extract with Soluplus^®^ (BG-SD SP) **(C)**, and spray-dried BG extract with PVP (BG-SD PVP). **(D)** Asterisks on bars indicate a statistically significant difference with respect to the untreated control, and asterisks on brackets indicate differences between the BG formulations and the excipients. *p ˂ 0.05, **p ˂ 0.01, ***p ˂ 0.01, and ****p ˂ 0.01.

The antimicrobial properties of the BG formulations were tested *in vitro*. Figure 1 shows the effect of the formulations on bacterial proliferation. Only BG-126 (−95.0% at 1,024 μg/ml catechin and 7.4% ethanol) and BG-SD (−105.6% at 512 μg/ml catechin) (Figures 1A,B) caused complete inhibition of bacterial proliferation equivalent to that of 1 μg/ml of tobramycin sulfate (positive control; not plotted). When comparing these MICs, BG-SD showed the highest potency. When expressed as verbascoside content, the MIC of BG-126 is equivalent to 513 μg/ml, while that of BG-SD is 333 μg/ml.

BG-126 also showed an increase in bacterial proliferation at low concentrations (≤8 μg/ml catechin and 0.06% EtOH). The MIC for BG-SD SP and BG-SD PVP could not be determined at the tested concentrations (Figures 1C,D). BG-SD SP reached a maximum inhibition of −70.1% (at 1,024 μg/ml catechin and 25.8 mg/ml SP), and BG-SD PVP reached −40% (near 128 μg/ml catechin and 3.2 mg/ml).

For the tested excipients, ethanol (Figure 1A) and PVP (Figure 1D) did not impact bacterial proliferation. On the other hand, SP did show the opposite effect, causing an increase in bacterial proliferation at a lower concentration (1–128 μg/ml catechin) and inhibition at 1,024 μg/ml (−91%).

### 3.3 Determination of the effect of the *Buddleja globosa* Hope formulations on *Pseudomonas aeruginosa* biofilm formation

**FIGURE 2:**
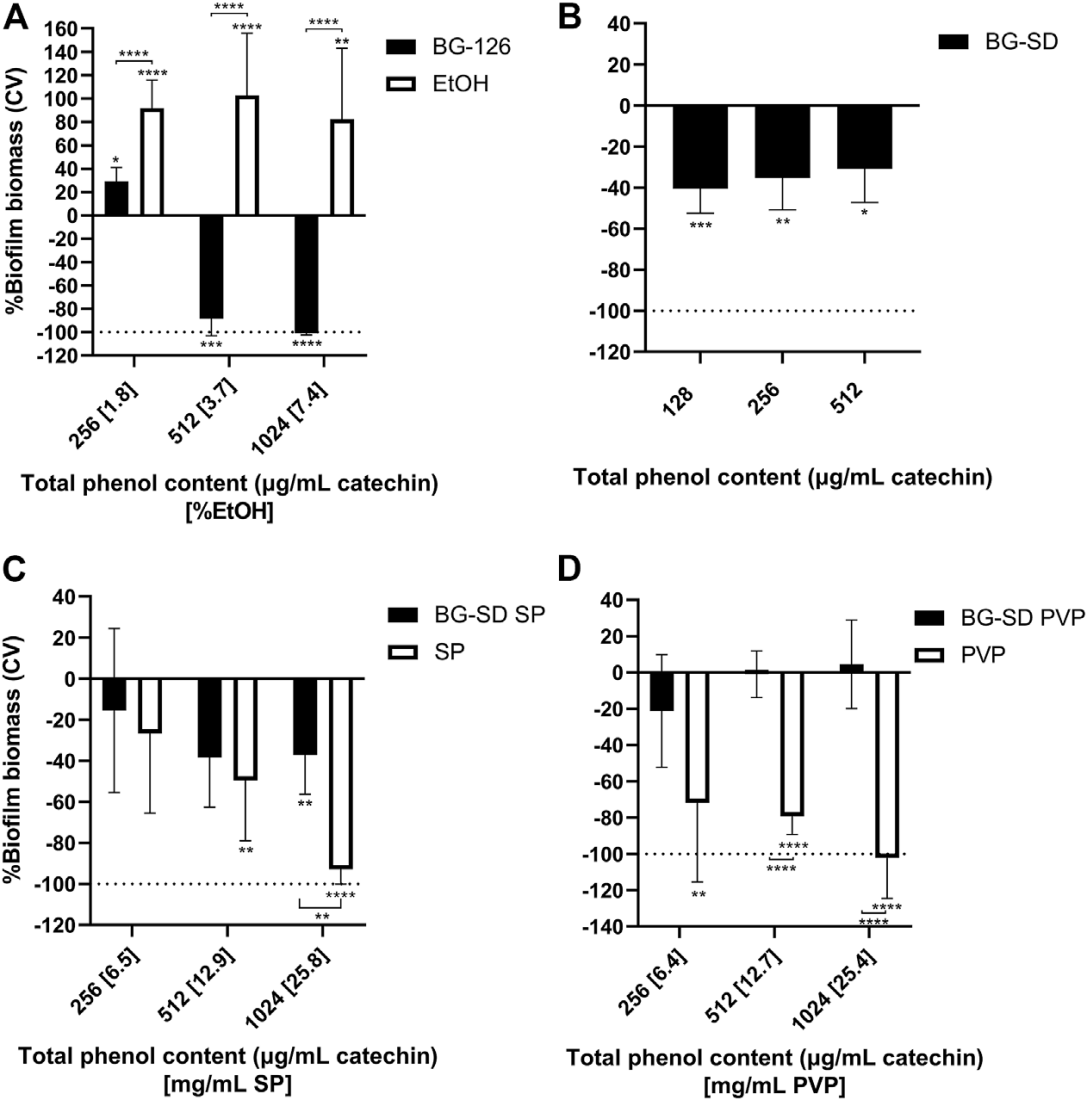
Effect of *Buddleja globosa* Hope (BG) extracts on *Pseudomonas aeruginosa* biofilm formation. *P. aeruginosa* was treated for 24 h with a hydroalcoholic BG extract (BG-126) **(A)**, spray-dried BG extract (BG-SD) **(B)**, spray-dried BG extract with Soluplus^®^ (BG-SD SP) **(C)**, and spray-dried BG extract with PVP (BG-SD PVP) **(D)** were stained with crystal violet (CV) for the determination of the biomass of biofilms based on the amount of CV retained by the stained wells. The results are expressed as changes in %biomass with respect to the control group. Asterisks on bars indicate a statistically significant difference with respect to the untreated control, and asterisks on brackets indicate differences between the BG formulations and the excipients. *p ˂ 0.05, **p ˂ 0.01, ***p ˂ 0.01, and ****p ˂ 0.01.

After 24 h of incubation of *P. aeruginosa* with the different BG extracts and the studied excipients, the microplates were stained with crystal violet for biofilm biomass determination. BG-126 inhibited biofilm formation completely at the determined MIC (equivalent to a total phenolic content of 1,024 μg/ml catechin and 7.4% ethanol) (Figure 2A). At the MIC of BG-SD, it reached about 40% inhibition of biofilm formation (Figure 2B). Similar inhibition was achieved with BG-SD SP (Figure 2C). BG-SD PVP did not affect *P. aeruginosa* biofilm formation (Figure 2D).

On the other hand, the tested excipients showed significant effects on biofilm formation (Figure 2, white bars). SP and PVP caused significant reductions in *P. aeruginosa* biofilm formation (Figures 2C,D), while ethanol caused a highly significant increase in biofilm formation (Figure 2A).

## 4 Discussion

### 4.1 Spray-dried *B. globosa* extract showed the highest potency to inhibit *P. aeruginosa* proliferation, while the hydroalcoholic extract BG-126 caused complete inhibition of biofilm formation

Using the broth microdilution method, we were able to determine the MIC for BG-126 and BG-SD against *P. aeruginosa*. These MICs were reported in terms of the total phenolic content expressed as the concentration of catechin. BG-SD caused bacterial inhibition at half the concentration needed for BG-126 (Figure 1). To the best of our knowledge, this is the first report on the inhibitory effect of a BG extract against *P. aeruginosa*. Mølgaard et al. tested the antimicrobial activity of BG and other methanolic extracts against seven microorganisms (including four strains of *P. aeruginosa*), but the authors did not observe relevant results with BG in this or other species (Molgaard et al., 2011). We also tested biofilm formation by BG-treated *P. aeruginosa* by crystal violet staining. BG-126 showed the highest potency to prevent biofilm formation (Figure 2A). The prevention of biofilm formation is an important attribute of a formulation aimed to treat chronic wounds. A high percentage of these wounds are colonized by biofilm-growing microorganisms such as *P. aeruginosa* (James et al., 2008).

It is essential to highlight that the antimicrobial properties of a natural extract can be a result of the activity of specific compounds and synergistic interactions between said compounds. Here, we used catechin to standardize the BG extract concentrations (Letelier et al., 2012; Letelier et al., 2017; Zamorano-Aguilar et al., 2020). This flavonoid has shown antimicrobial properties and synergism with antibiotics against Gram-positive and Gram-negative bacteria (Gomes et al., 2018; Ibitoye and Ajiboye, 2019; Zhang et al., 2021), and the reported MIC of pure catechin against *S. aureus* was around 78–156 μg/ml (Zhang et al., 2021). However, to date, there are no reports on the antimicrobial activity of catechin against *P. aeruginosa*, but there are reports on their effect against biofilm formation and proliferation (Lekbach et al., 2019). On the other hand, other BG components have been tested in *P. aeruginosa* cultures, showing inhibitory activity. Apigenin is probably the compound with the highest antimicrobial evidence of its inhibitory effect against *P. aeruginosa* (Basile et al., 2000; Ozcelik et al., 2011; Liu et al., 2013; Nayaka et al., 2014; Lucarini et al., 2015)*,* followed by quercetin (Basile et al., 2000; Ozcelik et al., 2011; Jaisinghani, 2017b; Wang et al., 2018) and linalool (Silva et al., 2015; Abd El-Baky and Hashem, 2016; Liu et al., 2020). Therefore, the antimicrobial effect exerted by the BG formulations might result from the activity of the combination and synergisms of different compounds such as apigenin, quercetin, and linalool and not by only catechin (Caesar and Cech, 2019). We used catechin as a referential compound to dose comparable concentrations of the extract. Additionally, we determined the verbascoside content for BG-126 and BG-SD. We did not express the concentration as mass/volume since the formulations with excipients (BG-SD PVP and BG-SD SP) would have a comparatively low concentration of the BG extract due to the diluting effect of the solubilizers in the formulation.

Dry powder formulations can be advantageous as powders have higher stability than solutions. Therefore, these spray-dried BG formulations have the potential to present higher stability. Spray drying has been used to encapsulate natural extracts showing great stability when stored in closed containers at room temperature (Cortes-Rojas et al., 2016). Additionally, these dry powder formulations can be used within capsules for oral administration (Letelier et al., 2017), to study their performance for inhalation delivery (Shetty et al., 2020), or used as ingredients for solutions or semisolid antimicrobial formulations for future applications. However, considering the poor flowability exhibited by the formulations, this property should be optimized for dosage forms that require fast filling steps, such as hard capsules or tableting, to prevent dose variability.

On the other hand, one of the disadvantages of spray drying is the low yield of recovery. Most of the powder is recovered from the collection vessel. However, there is a loss of powder that deposits along the system (drying chamber, connectors, cyclone, and filter). Since the commercial hydroalcoholic extract was obtained directly from the plant material, we could not quantify the yield in mass, but we obtained a high yield expressed as catechin content (64%–87%). Additionally, some loss of the material can be attributed to thermal degradation. Encapsulation of antioxidants has been shown to protect the integrity of compounds such as catechin and epigallocatechin gallate from thermal stress (Peres et al., 2011; Ho et al., 2017; Cruz-Molina et al., 2021). In our study, we used PVP as the encapsulating agent for the BG extract, and this BG-SD PVP formulation showed the highest yield, which could be attributed to the protective effect of PVP (da Fonseca Machado et al., 2018).

### 4.2 Tested excipients had significant effects on *P. aeruginosa* proliferation and biofilm formation

We used PVP (polyvinylpyrrolidone) and SP (Soluplus^®^) as solubility enhancers to improve the water solubility of spray-dried BG extracts. PVP is widely used as a pharmaceutical excipient, as a solubilizer to enhance the dissolution of drugs with low water solubility, and as a coating agent (Rowe et al., 2009). Soluplus^®^ is the commercial name of a co-polymeric solubilizer with an amphiphilic structure capable of forming colloidal micelles. Using PVP and SP, we reached higher testing concentrations in terms of the total phenolic content compared to BG-SD but without the ethanolic content of BG-126.

Previously, we found that pharmaceutical excipients, while considering inactive ingredients, may present antimicrobial effects that can synergize with antibiotics against biofilm-growing bacteria (Bahamondez-Canas and Smyth, 2018). Some pharmaceutical excipients, such as citric acid, succinic acid, glutamic, xylitol, and EDTA, present antibiofilm, bactericidal, and biofilm dispersion activities (Arias-Moliz et al., 2008; Badet et al., 2008; Ammons et al., 2011; Sommerfeld Ross and Fiegel, 2012; Khayyat et al., 2021). These excipients could also potentiate the activity of antibiotics (Allison et al., 2011; Sommerfeld Ross et al., 2017; Bahamondez-Canas et al., 2018). These authors proposed that these ingredients could act as nutrients reversing the quiescent state of a biofilm subpopulation that makes bacteria more tolerant to antimicrobials, may induce the dispersion of biofilms, or may prevent bacterial adhesion to surfaces. Therefore, in this study, we also tested the effect of these excipients alone on *P. aeruginosa* proliferation and biofilm formation. We observed that PVP significantly reduced biofilm formation (Figure 2D). PVP has been studied as a coating for prosthesis and tympanostomy tubes, showing the prevention of *S. aureus* and *P. aeruginosa* biofilm formation, respectively (Wolter and Hellstrom, 2004; Antonelli et al., 2011). Therefore, PVP could act as a coating agent on the surface of the multiwell plates used in these studies. However, we found no significant effect on biofilm formation with BG-SD PVP, indicating that the BG extract components might hinder this inhibitory effect of PVP. We also found that SP inhibited *P. aeruginosa* proliferation at 25.8 mg/ml (Figure 1C) and caused significant inhibition of biofilm formation from 12.9 mg/ml (Figure 2C), while BG-SD SP caused a moderate inhibitory effect at these concentrations (512 and 1,024 μg/ml catechin) (Figure 2C). These results indicate that SP can significantly reduce biofilm formation below the inhibitory concentration (25.8 mg/ml). This inhibitory effect of SP on biofilms has been described against *Staphylococcus epidermidis* when used as a polymeric nanocarrier (Takahashi et al., 2015; Albayaty et al., 2019; Takahashi et al., 2021). The amphiphilic nature of SP might contribute to this inhibition of biofilm formation as certain surfactants are known to weaken the biofilm matrix, causing biofilm disruption (Nguyen et al., 2020). Therefore, these excipients are good candidates to continue exploring the biofilm inhibitory effects in formulations.

Finally, we found that ethanol induced biofilm formation (Figure 2A). The treatment for 24 h with ethanol resulted in higher biofilm biomasses than the untreated control. Ethanol is well known as a disinfectant, which could be a desirable property but it has cytotoxic effects on mammalian cells (Farkas et al., 2003; Calderon-Montano et al., 2018; Kar et al., 2021). On the other hand, studies have shown that low ethanol concentrations (1 or 2%) are considered stress factors that induce biofilm formation (Chaieb et al., 2007; Tashiro et al., 2014) by increased cell aggregation (He et al., 2022) and induction of exopolysaccharide production (Chen et al., 2014). Our results using ethanol from 1.8 to 7.4% are correlated with this evidence. We also observed that diluted BG-126, containing 1.8% ethanol, caused an increase in biofilm formation with respect to the untreated control. This result could be attributed to the biofilm induction caused by a low ethanolic content, which might counteract the inhibitory effect of the BG-126 extract observed at higher concentrations. On the other hand, none of the three SD formulations showed induction of biofilm formation, indicating that the residual ethanolic content in the powders is not significant, which is, indeed, another advantage of formulating BG extracts by spray drying. The residual solvent content of spray-dried powders is usually below 5% (Bahamondez-Canas et al., 2018). Da Fonseca Machado compared three techniques for PVP encapsulation of natural ethanolic extracts finding that spray drying resulted in the lowest residual ethanolic content (2%) when compared to freeze drying (5%) and supercritical antisolvent (8%) (da Fonseca Machado et al., 2018).

### 4.3 Formulation with the excipients PVP and SP improved the water solubility of the spray-dried BG extract at 37°C

The improvement in the water solubility was the first aim of this research to achieve higher concentrations of the actives without the ethanolic content. As expected, we found a low aqueous solubility for BG-SD at 25°C that increased with the addition of excipients and at 37°C (Table 2). Determining the solubility at 37°C was necessary due to the characteristics of the wound site as the actives intended for wound healing must be able to dissolve in the wound fluid at body temperature. The wound fluid or exudate represents a small volume of liquid below 6 ml (Gohel et al., 2008), with reports on exudate production of 0.2 ml/h for pressure ulcers (Iizaka et al., 2010). Therefore, improving the solubility of the BG components is critical to exploiting their antimicrobial properties for wound care.

To determine the solubility of a compound, solubility studies are routinely performed during preformulation studies. This determination is achieved by adding an excess of a compound in a fixed volume (25–50 ml), followed by agitation at room temperature that can last several days. This study is conducted using water and different buffers to fully characterize the solubility of a said active ingredient (Bonfilio et al., 2018). In our research, we evaluated the antimicrobial properties of the BG extract based on the evidence of its wound healing properties (Backhouse et al., 2008; Bustamante et al., 2015). Therefore, with this application in mind and the incubation temperatures needed for antimicrobial testing, we also conducted this solubility test at a physiologically relevant temperature of 37°C and in smaller volumes. The United States Pharmacopeia describes the solubility of a compound, regardless of the solvent used (Savjani et al., 2012). All SD formulations can be classified as very slightly and slightly soluble. When comparing solubilities in terms of the total phenol content expressed as catechins, then the SD formulations can be described as freely to very soluble. Cuevas-Valenzuela et al. determined that the water solubility of catechin increases with temperature and ethanol concentration (Cuevas-Valenzuela et al., 2014), supporting the relation between solubilities we obtained for BG-SD and BG-126.

## 5 Conclusion

The BG extract obtained by spray drying (BG-SD) was capable of inhibiting *P. aeruginosa* proliferation with a higher potency than the hydroalcoholic extract BG-126. On the other hand, BG-126 prevented biofilm formation and the excipients PVP and Soluplus^®^ alone. We also observed that low concentrations of ethanol alone or in BG-126 induced *P. aeruginosa* biofilm formation. Although the spray-dried BG extracts with excipients showed lower antimicrobial efficacy than BG-126 or BG-SD, these BG formulations can be used to investigate biocompatible topical therapies for wound healing as non-ethanolic alternatives. Spray drying allowed the development of a water-soluble BG extract with promising antimicrobial efficacy for further research and development of novel phytopharmaceuticals to treat *P. aeruginosa* infections.

## Data Availability

The raw data supporting the conclusion of this article will be made available by the authors, without undue reservation.
