# Supplementary figures and images for: Formulation of water-soluble Buddleja globosa Hope extracts and characterization of their antimicrobial properties against Pseudomonas aeruginosa
Source: Front Pharmacol. 2022 Nov 10;13:921511. doi: 10.3389/fphar.2022.921511 (PMC9685521; doi:10.3389/fphar.2022.921511)

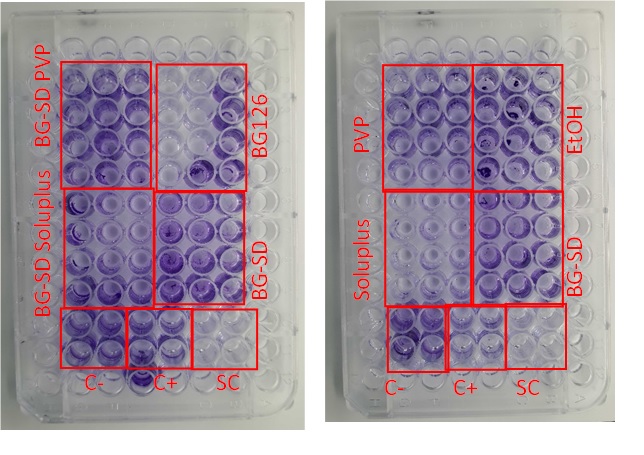

Supplement: Supplementary file 1 [file Image1.JPEG]
